# Sexual Assault of Women in the region of Kairouan, Tunisia: an 8-year retrospective study on epidemiological and medicolegal characteristics

**DOI:** 10.1186/s12905-022-01647-8

**Published:** 2022-03-08

**Authors:** Oumeima Brahim, Elyes Turki, Elaa Chebbi, Oumayma Fersi, Ridha Fatnassi

**Affiliations:** 1 Department of Legal Medicine, Faculty of Medicine of Sousse, Ibn El Jazzar Teaching Hospital of Kairouan, 3100 Kairouan, Tunisia; 2 Department of Obstetrics and Gynaecology, Faculty of Medicine of Sousse, Ibn El Jazzar Teaching Hospital of Kairouan, 3100 Kairouan, Tunisia

**Keywords:** Sexual assault, Female victims, Pattern, Rape, Forensic examination, Forensic evidence collection

## Abstract

**Background:**

Despite the abundance of studies reporting the prevalence of women's sexual abuse all over the world, there is a real lack of such reports in developing countries in general and Arab-Muslim societies in particular. However, due to the little number of published studies in Tunisia, and the absence of a national database, data on female sexual assaults are still underestimated, which is a gap that needs to be filled in order to make specific preventive actions. We aim to identify the pattern of female victims of sexual abuse in the governorate of Kairouan (Tunisia) in order to provide recommendations for prevention.

**Methods:**

Retrospective data were collected on all-female sexual assault victims, particularly rape, presented to the Department of Forensic Medicine of the University Hospital Ibn El Jazzar of Kairouan (Tunisia), during an 8-year period, from 2009 to 2016.

**Results:**

Two hundred and sixteen victims were included. Age ranged from 3 to 82 years with a mean age of 20.4 years. Victims were single in 84.3%, unmarried in 90.7% and they lived in rural areas in the majority of cases. Rape was committed by a single individual in 94.9% of cases, and the assailant was a stranger in only 26.8%. The assault occurred most frequently in the assailant's home (73.6%) or the public places (11.6%). Evidence of recent acute general body trauma was found in 41.2% of the victims, and the most common injuries were located on the thigh, upper arm, and chest. In 28.1% of the cases, injuries were seen in the face and the neck. Genital examination showed that 188 victims (87%) had a tear in the hymenal membrane and only 13% of victims had intact hymenal membrane. A complacent hymen was noted in 2.3%. A recent anal lesion was seen in 8.3% of the cases. Cytology was performed on 78 victims. In 22 cases (28.2%), sperm could be detected in vaginal swabs up to 3 days post-assault, and pregnancy was seen in 7.4% of assault victims.

**Conclusion:**

Sexual abuse represents a human rights and public health problem that is thriving in a culture of silence, particularly in the Arab region. There is a particular need to create a Tunisian national database on female sexual assaults, in order to centralize data and provide holistic follow-up for specific preventive measures. Finally, efficient management of such cases will need, in addition to legislation, a partnership between the various actors involved in taking care of the victims (health care professionals, the police, social specialists, and psychologists). In addition, civil societies are key partners to break the silence, support this issue, and raise awareness.

## Background

Sexual violence is a widespread public health issue with a wide range of legal, medical physical, psychological, and social aspects. It is a hard and violent fact for millions of victims in the world, mainly women [1]. Given the upsetting consequences of this problem, several studies from different countries have been published to analyze its epidemiological aspects and to set up further preventive measures.

According to the world health organization (WHO), one in three women suffers from sexual abuse (SA) at least once in a lifetime [1, 2]. In the US, every 98 s one person is sexually abused, among which 90% are women [3]. In France, according to a study conducted in 2017, sexual assault occurs to 600,000 women per year [4].

Despite the abundance of studies reporting the prevalence of sexual women abuse all over the world, there is a real lack of such reports in developing countries in general and Arab-Muslim societies in particular [5, 6]. Little attentiveness has been paid recently to violence against women in the Arab countries given its devastating consequences [6]. For example, during the period between 2003 and 2008, a clear increase in the prevalence of violence against women was noted in Morocco, raising from 1.3 to 6.2 cases per 100,000 women [6].

The extent of women abuse in Arab countries can be explained by multiple sociocultural factors, such as stigma, notions of male authority embedded in traditional culture, and the misuse of religious rules [7, 8].

The measurement of sexual assault is one of the most critical challenges in the field of women abuse research, especially in Arab-Muslim countries. Diagnosis of SA is not always obvious and the doctor can refer to further examinations [9].

In Tunisia, little data are available about the epidemiology of women SA. A report was published by The National Office for Family and Population in 2011, showed that roughly one in six women (16.7%) aged between 18 and 64 years old have been a victim of sexual abuse at least once in their lives [10]. Another national study was conducted by the Center for Research, of Documentation and Information on Women (CREDIF) found that more than one in two women (53.5%) have been victims of violence in public spaces in the last 4 years (2011–2015), of which 75.4% were sexual violence [11].

An Emergency Forensic Unit (EFU) was created on March 08, 2016 at Charles Nicolle Hospital to welcome and to take care of assaulted women who already initiated legal actions and who are examined based on a judicial requisition. This Unit covers four governorates of Tunis’s metropole, which represent about 23.2% of the total population with an average of 650,000 per governorate. A recent study was conducted by Ben khelil et al., to identify the profile of female victims of sexual assaults committed in the Metropole of Tunis over a period of 13 months (between 2016 and 2017), has reported that female victims represented 77% of cases of sexual assault [6].

Real incidence may be higher in Tunisia, as lack of declaration of violence is common as a consequence of fear of society, shame, and lack of information about legal rights. Under-reporting is more important in the case of SA because it remains strongly stigmatized [7, 8].

However, due to the little number of published studies and the absence of a national database, data on female sexual assaults are still underestimated in Tunisia, which is a gap that needs to be filled in order to make specific preventive actions.

Our study aimed to identify the pattern of female victims of SA, particularly rape, on Tunisian sample in order to provide recommendations for prevention.

## Methods

The current survey is a retrospective descriptive analysis of forensic examinations in female sexual assault victims, particularly rape committed in the Governorate of Kairouan (Tunisia), who consulted in the Department of Legal Medicine of the University Hospital Ibn El Jazzar of Kairouan, in an 8-year period, from 2009 to 2016.

The governorate of Kairouan is located in the center of Tunisia, which has an average population of 570,436 in 2014, 5,2% of the total population of Tunisia with low urbanicity (33%) [12].

We included the medical-forensic examination of all the female victims of SA, particularly documented rape with physical and/or biological proof. Cases of sexual assault other than rape were excluded. We also excluded the cases of refusal of examination and male sexual assaults.

This study was particularly interested in rape given the importance of the virginity concept in the Arab-Muslim societies in general and Tunisian society in particular. Virginity in our culture is proved by an intact hymen.

Was considered physical evidence of rape, all types of traumatic injuries in the genital and anal areas. Pregnancy was also considered as physical evidence.

Was considered biological evidence of rape, the detection of semen on the victim's body.

The examination of victims followed the World Health Organization's recommendations for the forensic management of victims of sexual violence [13].

Before being examined, the victims’ informed consent was obtained. The victim's interview followed the World Health Organization's safety and ethical guidance for carrying out research on Domestic Violence Against Women [14]. To identify abusive sexual behavior, women were directly questioned using a discussion guide. The medico-legal examination was assisted by at least two forensic doctors. A detailed “top-to-toe” physical examination of the victim was conducted in order to look for stigmata of a traumatic nature [13].

The genito-anal examination was performed in the lithotomy position (the patient being placed lying on her back with her knees drawn up, heels together, and legs gently flopped apart).

The complacent hymen is defined as a dilatable one, which can be extended without being injured during sexual intercourse. The medico-legal expertise allowed to detection of the complacent hymen by the introduction of two gloved and oiled fingers without damaging the hymeneal tissue. Also, hymen which could be distended up to three centimeters without damage is considered complacent hymen [15]. It is the examination of the hymen that will enable us to say if there has been defloration or not. The hymen is the anatomical evidence of defloration.

The anus was initially inspected and gentle buttock traction was sustained for 30 s to allow anal dilatation to proceed when present. A rectal digital examination was performed to evaluate the tone of the anal sphincter.

After having eliminated by interrogation the other possible causes that can induce anal injuries, recent anal injuries due to rape were used to describe the following lesions: Swelling and erythema (reddening) of the perianal tissues, Venous congestion, Fissures, abrasion, and bruises [16, 17].

Based on previous studies, old and healed anal injuries which evoke signs of recurrent anal penetration were used to describe the following lesions: Reduced tone of the anal sphincter and laxity, Gaping, Anal dilation, Funnelling, and Scars [16, 17].

Any biological evidence was collected with moistened swabs (for semen).

Cases with a delayed menstrual period and suspicion of pregnancy were referred by the Department of Forensic Medicine to Gynecology-Obstetrics Departments for further explorations and examinations (BHCG testing and obstetrical ultrasound).

All data were obtained by the examination of medical report sheets applied by forensic doctors, that were used in cases of suspected sexual assault at the request of the police.

Data of interest were as follows: sociodemographics of the victim (age, socioeconomic and education level, social and psychological background, vulnerability factors); the assault characteristics and circumstances (date, time, place, type of the assault including vaginal, anal, time from assault to the medical examination and use of physical force); assailant characteristics (age, gender, levels of relationship to the victim); findings at the physical examination (the presence of bodily, genital and/or anal injuries); medico-legal intervention ( genetic findings including presence of sperm, obstetrical explorations including a pregnancy test and echographic and detection of sexually transmitted infections).

The statistical analysis was performed by using IBM SPSS version 23 for windows.

In Tunisia, specific legislation was approved in 2017 to spread gender equality and prevent all types of violence against women and particularly sexual assault and child abuse, which was used as a basis for the definition of some parameters in this work [18].

Sexual assault is defined by this law as “any act or declaration aimed at subjecting the women to their sexual needs or those of others through the use of defamation, coercion, force and other ways of weakening and stripping the will, regardless of the victims' relationship.”

Is considered as rape according to the same law “any act of sexual penetration, regardless of the type and the means used, committed on a female or male person without her consent”.

Situations of vulnerability were defined by this law as “the situation of fragility linked to young or advanced age, serious illness, pregnancy, or mental or physical deficiency affecting the victim's ability to resist the perpetrator”.

We have considered as vulnerable a person who is legally unable to consent, i.e., under the age of 16 years, according to the law n° 2017-58. This study uses the WHO's standard of age 60 to describe “older” people [19].

## Results

### The victim’s characterization

During the period of the study, a total of 489 cases of sexual abuse were examined. Female victims, represent 90.18% (n = 441). 216 cases fulfilled the inclusion criteria (48.9%), comprising the sample of our study. Table 1 summarises the victim sociodemographic characteristics of the cases.

**Table 1:** Socio-demographic characteristics of assaulted victims

Variable	Number	Percentage (%)
Origin		
Rural	122	56.5
Urban	94	43.5
Socioeconomic level		
Low	149	69
Moderate	67	31
High	0	0
Education level		
Illiterate	21	9.7
Primary school	89	41.2
High school	104	48.2
University	2	0.9
Occupation		
Unemployed	146	67.6
Student	17	7.9
Day laborer	39	18
State official	14	6.5
Age groups		
< 13 years	9	4.1
13–17 years	118	54.7
18–24 years	48	22.3
25–35 years	25	11.5
> 35 years	16	7.4

The victims’ ages varied between 3 and 82 years old with a mean age of 20.4 years.

The majority of the victims were children aged under 18 years (n = 127; 58.8%). Young women (18–35 years old) counted for 33.8%.

Victims were from a rural area in 56.5% (n = 122) and they had a low socio-economic level in 69% (n = 149). They were single in 182 cases (84.3%), and unmarried in 196 cases (90.7%).

The majority of victims were with an educational level of high school (n = 104; 48.2%) and only 21 victims were illiterate (9.7%).

In more than half of the cases, the victims were unemployed (n = 146; 67.6%). They were day laborers in 18% (39 cases), and students in 7.9% (17cases). Table 1 presents the various descriptive information regarding victim sociodemographic characteristics.

**Fig. 1:**
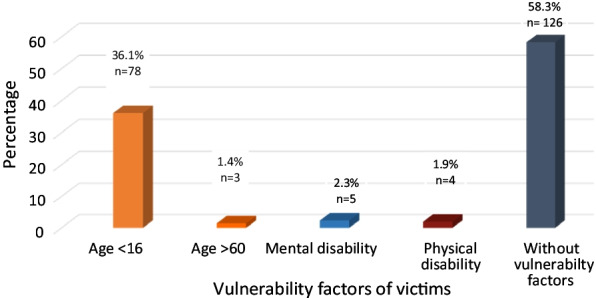
The vulnerability factors of the victims

Ninety victims had vulnerability factors (41.7%), including age under 16 years old in 87 cases (36.1%), a mental disorder in 5 cases (2.3%), and physical disability in 4 cases (1.9%) as illustrated in Fig. 1.

### History of assault

Assaults were committed in rural areas in 56.5% (122 cases). Table 2 shows that the assault occurred most frequently in the assailant's home (73.6%) or the public places (11.6%).

**Table 2:** Place of assault, forensic medical examination, and evidence findings

Variable	Number	Percentage (%)
Place of the assault		
Assailant’s house	159	73.61
Victim’s house	24	11.11
Public place	25	11.57
Workplace	5	2.31
Other	3	1.39
General body trauma	89	41.2
Type:		
Bruises	63	70.8
Superficial abrasions	46	51.7
Cigarette burns	5	5.6
Wounds	4	4.5
Location:		
Thigh	61	68.5
The upper arm	43	48.3
Chest/breasts	28	31.5
Face and neck	25	28.1
Genital findings		
Old hymeneal transection	150	69.4
Recent hymeneal transection	38	17.6
Complacent hymen	5	2.3
Injury to the anal area	38	17.6
Recent anal injury	18	8.3
Old anal injury	20	9.3
Forensic collection and evidence finding		
Vaginal sperm detection	22	28.2
Pregnancy	16	7.4

The majority of cases took place during the spring months (40%), followed by summer and autumn, 23% and 21% respectively. The highest number of cases was recorded in March (17.3%) and April (12.3%). Whereas, 14.2% of cases were reported in September.

In 139 cases (64.4%), assaults were observed in the second half of the week. It was seen on Thursday with 20%, followed by Saturday and Friday, 16.5% and 16% respectively.

**Fig. 2:**
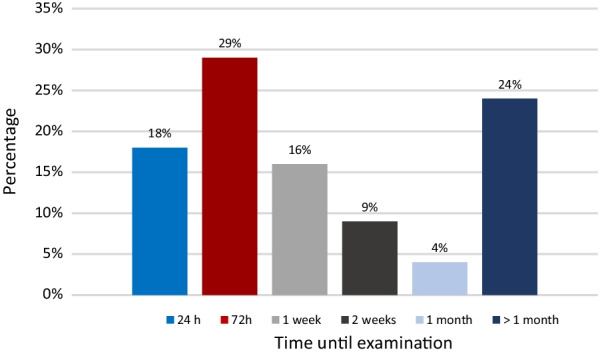
Time until the examination

101 victims (46.8%) were examined within 72 h of the date of the assault. A total of 136 medical-forensic examinations (63%) took place within the first-week post-assault (Fig. 2).

### Suspect identification

The assault was committed by a man in all cases. The perpetrators’ ages varied between 12 and 70 years old with an average age of 25.7. The majority of the cases were committed by adults aged 18 years and older (n = 207; 95.83%). In 9 cases (4.16%), the assailant was an adolescent, aged under 18 years old. The highest number of cases being recorded by perpetrators in the age group of 18–30 years (n = 175; 81%) followed by the age group of 30–40 years (n = 27; 12.5%).

In 94.9% (205 cases), the sexual intercourse was committed by one individual versus 7 cases (3.2%) was a group rape committed by 2 or 3 individuals. In 4 cases (1.9%), the number of perpetrators was greater than 3 with 07 assailants in one reported case.

**Fig. 3:**
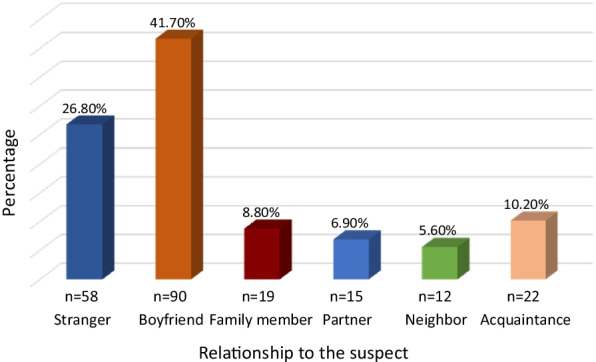
Relationship to the suspect

Data related to the relationship between perpetrators and victims showed that assailants were strangers to the victim in 58 cases (26.85%), while they were acquaintances in 158 cases (73.15%). Among the latter group, 41.7% were the victim’s boyfriend (n = 90), 6.9% were the victim’s partner (fiance or husband) (n = 15) and in 5.6% of the cases (n = 12), the assailant was the victim’s neighbor (Fig. 3).

In 8.8% (n = 19) of the cases, the alleged assailant was a family member. An incestuous relationship was noted in four cases (1.85%) and the perpetrator was the father in one case, the brother in one other case and it was a paternal uncle in two cases. In 10.2% of the assault cases (n = 22), the relationship between the assailant and the victim was just a simple acquaintanceship (Fig. 3).

### Physical examination findings

Table 2 summarizes the medical (general body findings, genital and anal injuries), and laboratory findings of the cases. Evidence of recent acute general body trauma was found in 89 cases (41.2%). Bruises were the most frequent type of injuries, seen in 63 cases with 70.8%, followed by superficial abrasions seen in 46 cases (51.7%). Cigarette burns and wounds were seen in 5.6% and 4.5% respectively.

The most common injuries were located on the thigh in 68.5% of the cases (n = 61), upper arm (48.3%; n = 43), and chest/breasts (31.5%; n = 28). In 28.1% of the cases (n = 25), injuries were seen in the face and the neck.

Genital examination showed that 188 victims (87%) had a transection in the hymenal membrane, and only 28 victims (13%) had intact hymenal membrane. A complacent hymen was noted in 5 cases (2.3%). In 150 cases (69.4%), old transections were noted, suggesting previous intercourse. While in 38 cases (17.6%), one or more recent complete transections of the hymen were seen. In 16% of the cases, victims had one hymenal tear and in 19%, double hymenal tears were seen. Multiples tears (more than three transections) were identified in the majority of genital examinations with 65%. Transections were in the majority of the cases located in the posterior fourchette (n = 155; 82.5%). The most frequent localization of the hymenal transections was recorded between 3 and 9 o'clock, and between 5 and 7 o'clock.

17.6% of victims (n = 38) had at least one injury to the anal area of which 28 cases had only anal injuries without hymenal transection. A recent anal lesion was seen in 8.3% of the cases (n = 18), whereas old and healed anal lesions were found in 20 victims (9.3%), which evoke signs of recurrent anal penetration. The presence of both recent and old lesions to the anal area was seen in 5 examined victims.

### Forensic collection and evidence finding

During the medical-forensic examination, vaginal swabs were taken from 78 victims (36%) and were afterward examined for evidence of sperm. Of 78 victims, vaginal sperm could be detected in only 22 cases (28.2%), whereas sperm detection was unsuccessful in 56 victims (71.8%). No sperm could be detected 3 days post-assault.

Pregnancy following sexual abuse was seen in 16 cases (7.4%). It was screened by a dosage of Beta HCG in 13 cases (6%) and then confirmed in all the cases by an obstetrical ultrasound (Table 2).

No cases of sexually transmitted diseases have been found in the current study.

## Discussion

Medical-forensic examinations provide a basis for the documentation and interpretation of medical findings in the field of sexual assault that will not be found later [20]. Although it is widely accepted that is difficult to determine the real magnitude of such abuse due to the limited statistical data regarding this subject. Forensic investigators need to show more interest in this problem [21].

The statistics from high-income countries reflect a relatively high prevalence of SA. United States (US) National Intimate Partner and Sexual Violence Survey (NISVS) found that one in five women (18.3%) reported having been sexually assaulted in their lifetime compared with one in 71 men. The prevalence was highest according to South African studies, the prevalence rates of sexual assault was ranging between 12 and 28% of women [22].

Real incidence may be higher in Arab societies, as under-reporting of sexual violence remains common [7].

In Tunisia, little data are available about the epidemiology of sexual abuse. To the best of our knowledge, this study is the second one to address the victim profile of sexual abuse in Tunisia [6].

In the current study, the victims' pattern revealed that the majority of the females were not married, unemployed with low socio-economic levels, and had completed some or all of their high school education. Their mean age was 20.4 years and they had no known history of medical disease or mental illness. The victims were most frequently assaulted in the assailant's home during the spring months. The assailant was mostly known to the victim, either the victim's boyfriend or an acquaintance. The assault was reported almost within 3 days of the assault.

The principal limitations of this survey are that is a retrospective study, which makes some data not always clarified, and that our results could not be generalized to all Tunisian population given that we only worked on the Governorate of Kairouan (Tunisia). Moreover, our findings could be underestimated in terms of the rate of rape as we only took into account victims who had already started legal procedures.

In agreement with other studies conducted in American, European, African, and Tunisian countries [3, 6–8, 20, 23] the majority of the sexual assault victims were female (90.18%). The increased number of SA among females is due to gender-based discrimination that can take the form of violence, sexual abuse, and exploitation. Thus, the female gender is considered a risk factor for sexual abuse. But it should be kept in mind that sexual assault is less frequently reported by male victims [20].

Our study showed that most of the victims were single and young with a mean age of 20.4 which was comparable to another Tunisian study performed by Ben Khelil et al. [6] and another study that was performed in Egypt [24]. The average age in our survey was slightly less than the mean age found in other studies carried out in Marseille (South of France), Denmark, and Canada (25.8 years, 25, and 24.1 years respectively) [23, 25, 26] and above the mean age performed in Tours (France) [27]. These particular characteristics may be attributable to their lifestyle and increased susceptibility to being sexually assaulted. Some susceptibility to being assaulted may be linked to the fact that they are single, divorced, or separated, leading to undesired sexual behavior [6, 28].

Regarding the age of victims, we found that over half (58.8%) of sexual assault cases happened among patients aged 17 years and younger. These results are consistent with another Tunisian study, where Children presented 63.1% of victims [6]. In addition, an Egyptian study revealed that the highest percentage of assault cases was among females aged between 12 to 18 years [24]. A higher sexual assault rate among younger victims was reported by other French studies, e.g., Grossin et al. and Saint Martin et al., with a mean age of 15.9 years and 16.5 years respectively [9, 27].

Thus, young age is considered as a risk factor of SA and young girls are particularly vulnerable [28]. The tendency of younger women to report sexual abuse more frequently in comparison with older ones may be explained by the fact that sexual assault on children frequently takes place without the use of physical force. Also, it can be explained by the physical attraction to younger women [20].

In our survey, it was reported that the lowest percentage of victims was among females aged older than 35 years, which is consistent with an Egyptian study [24]. This may be due to the tendency of older females to be more protective of acquaintances and strangers.

In agreement with the World report on violence and health [29], the current study showed that 56.5% of sexual assault occurred in females who lived in rural areas and 69% had a low socioeconomic status. The fact that the majority of victims were unemployed is also consistent with the literature [6, 26, 30].

Rural areas are characterized by precarious conditions with difficulties of access to education and care. Economic need and lack of social support also play a major role in sexual exploitation [28].

In our study, five victims (2.3%) had a mental illness and another four had physical deficiencies (1.9%). These data coincided with an Egyptian study, which reported that mental illnesses were reported in a small number of victims [24]. According to a French study by Saint-Martin et al., 7% of the victims were having mental or physical deficiency and 4% were suffering from a psychiatric disorder [27]. Other studies report that up to 90% of disabled persons were victims of sexual violence [31].

In keeping with another Tunisian study [6] and with the literature [32, 33], we found that the majority of cases took place during the spring months (40%). This was not correlated with the Danish study, which showed that assaults occurred preferentially in summer [34].

A potential explanation might be linked to the religious month of Ramadan, which took place in the summer during the period of the study. This could lead to lower rates of sexual assault given its religious symbolism, that women spent the most time with relatives, and that there was significantly less alcohol consumption. Also, this increased prevalence of SA in spring may be explained by the coincidence with school holidays in Tunisia during which leisure activities and hanging out increase. Another explanation could be related to the fact that Testosterone levels can change according to the season, which can promote changes in physiology and human behavior, such as sexual intercourse [32].

Consistent with the literature [27, 34, 35], the present study showed that the perpetrator was someone known by the victim in the majority of cases (73.15%). This high rate of known assailants can be explained by the fact that the perpetrator is often a close acquaintance who lives in the same household or in the same residential area who is frequently in the victim`s home and who sometimes exerts legal authority.

In the current study, the assailant was a family member in 8.8% of the cases. Referring to a study carried out by Janish et al., the incidental rate of a family member being the perpetrator of SA, was 24.3% which is slightly more than double of our result [20]. 1.85% of all the SA cases in the present study were incest, these findings seem to be less than the incidental rate of incest in France (5%). This lower rate can be related to the very different traditional moral values and cultural backgrounds of various societies on the one hand and may also be explained by the fear and shame of disclosure in Arab societies on the other hand.

The current study showed that fifteen victims (6.9%) were sexually abused by their partners. The prevalence of sexual intimate partner violence was found to be higher in the WHO African and Eastern Mediterranean regions. The first worldwide systematic review (2013) regarding the prevalence of sexual violence between intimate partners in the Arab World, shows that 30% of all women have been physically and/or sexually abused by their intimate partner [7]. In the United States, 10% of women have been raped by an intimate partner (3 in 10 women). They are more likely to be raped by their intimate partners than by strangers [36].

A stranger was implicated in 26.82% of the sexual assault victims in the present study. This finding is similar to comparable studies, where stranger sexual assault varies from 23 to 40% [26, 37].

In accordance with other Tunisian and Egyptian studies, the majority of the assaults occurred in the assailant’s home (73.61%) [6, 24]. Riggs et al. and Saint-Martin et al. also reported that most of SA were recorded in the offender’s and victim’s home, which can be explained by the fact that the offender is often someone known to the victim [27, 37].

Forensic examination in the majority of the cases (63%) took place within the first-week post-assault, of which 45% were examined after 72 h. This is comparable with other studies [20, 21, 24, 26, 30]. About a quarter of victims (24%) consulted after one month of the SA event. Adama et al., reported that this delay can be explicated by fear, shame, embarrassment, and lack support from family [38].

In the current study, evidence of genital trauma was found in 87% of cases, which was more commonly documented than general body trauma (41.2%). Similarly, Grossin et al. found extra-genital injuries only in 39.1% [9]. The lower rate of general body injuries compared to genital injuries may be explained by the fact that the victims are often minors and vulnerable and that sexual assault on children commonly takes place without the use of physical force.

Similar to the published literature, [20, 21] bruises were the most frequent type of injuries with 70.8%, followed by superficial abrasions (51.7%), while the other forms of injury.

The most common injuries were located on thighs, upper arms, and chest/breast (68.5%, 48.3%, and 31.5% respectively). In 28.1% of the cases, injuries were seen in the face and the neck. A similar order of extragenital injuries was also reported by other studies [20, 21, 39].

Lesions observed to the neck region suggest the assailant’s attempt to exert control, while the arms and legs are to be regarded as self-defense injuries and suggest resistance from the victim.

In agreement with the published studies [9, 20], vaginal intercourse was the most common type of penetration (65.7%). Similar to other surveys, the posterior fourchette is the most common location for hymenal tears following sexual assault (82.5%) and especially between 5 and 7 h [23, 40]. However, we should keep in mind that the absence of recent genital injuries does not rule out the possibility of SA. Drug-induced assaults and non-consented sexual intercourse in the case of the complacent hymen are considered among the factors that may not leave clinical signs. Vaginal penetration on a previously torn hymen is another factor that can explain the absence of genital injury in case of rape. Moreover, genital findings are sometimes difficult to interpret such as vulvar and vaginal erythema, which can be found also in local infections [9]. The anal injury was observed in 38 cases (17.6%), as reported by Riggs et al. [37].

In our study, cytology was performed on 78 victims (36%) for evidence of sperm. Only in 22 cases (28.2%), vaginal sperm could be detected whereas sperm detection was unsuccessful in 56 victims (71.8%). These results are similar to the literature [9, 34, 37], which reported a successful sperm detection ranging from 15 to 45%. Our findings showed that no sperm could be detected 3 days post-assault, as reported by Jänisch et al. [20].

Negative findings in specimens from victims of sexual assault may be explained by several factors such as a prolonged post-assault interval, penetration without ejaculation, use of spermicidal agents, digital penetration, azoospermia, or oligospermia, and vaginal inflammation [9]. The varying results of sperm detection suggest that the protocol of examination in sexual assault needs to be optimized. Improvements could be achieved by the use of other techniques to detect sperm even after 48 h such as prostate-specific antigen-test [34].

The sexual assault-related pregnancy rate in the current case (7.4%) was slightly higher than the other studies [37, 39], which can be explained by the lack of use of pregnancy prophylaxis [30].

To summarize, sexual assault, represented a worldwide serious issue. However, due to the lack of published studies and the absence of a reporting system, the rate of SA could be under-estimated, which is a gap that needs to be filled in order to make specific preventive actions. To reduce this phenomenon, providing recommendations of a public health-based prevention strategy is needed.

Media and social campaigns may lead to a serious mass of revised social norms and attitudes. Also, the role of the Government and its capacity to defend the rights of sexual assault victims is very important. In fact, the existence of strong legislation may help in providing protection against female violence.

In Tunisia, policymakers have tried to spread gender equality by approving a new law in 2017 « on the elimination of violence to women». This law is considered as a solid legislative framework to prevent all types of violence against women and especially sexual assault and child abuse by rising the penal sanctions and offering psychosocial support and legal assistance [18].

Despite the existence of specific laws, the current study highlighted a relatively high number of female sexual assaults, which strengthens the need for multidisciplinary preventive approaches.

## Conclusion

Examination of sexual assault victims is one of the most serious challenges in the field of victimization research and is often underreported in developing countries and Arab societies. In order to reduce this worldwide problem, future research is still needed and specific preventive programs are needed to provide social support and care among vulnerable people and to eliminate gender inequalities.

## Data Availability

The datasets generated and/or analyzed during the current study are available from the corresponding author on reasonable request. Data available on request due to the data are not publicly available due to privacy and ethical restrictions (e.g. their containing information that could compromise the privacy of research participants).

## Abbreviation

SA: Sexual abuse
